# Supplementary material for: Frequency wavelength multiplexed optoacoustic tomography
Source: Nat Commun. 2022 Aug 1;13:4448. doi: 10.1038/s41467-022-32175-6 (PMC9343396; doi:10.1038/s41467-022-32175-6)
Supplement: Supplementary file 1 — Supplementary Information [file 41467_2022_32175_MOESM1_ESM.pdf]

Frequency Wavelength Multiplexed Optoacoustic Tomography

Antonios Stylogiannis[1,2], Ludwig Prade[1,2], Sarah Glasl[1,2], Qutaiba Mustafa[1,2], Christian Zakian[1,2], Vasilis Ntziachristos[1,2]

[1]Technical University of Munich, Germany, School of Medicine, Chair of Biological Imaging
[2]Helmholtz Zentrum München, Neuherberg, Germany, Institute of Biological and Medical Imaging

Supplementary Information

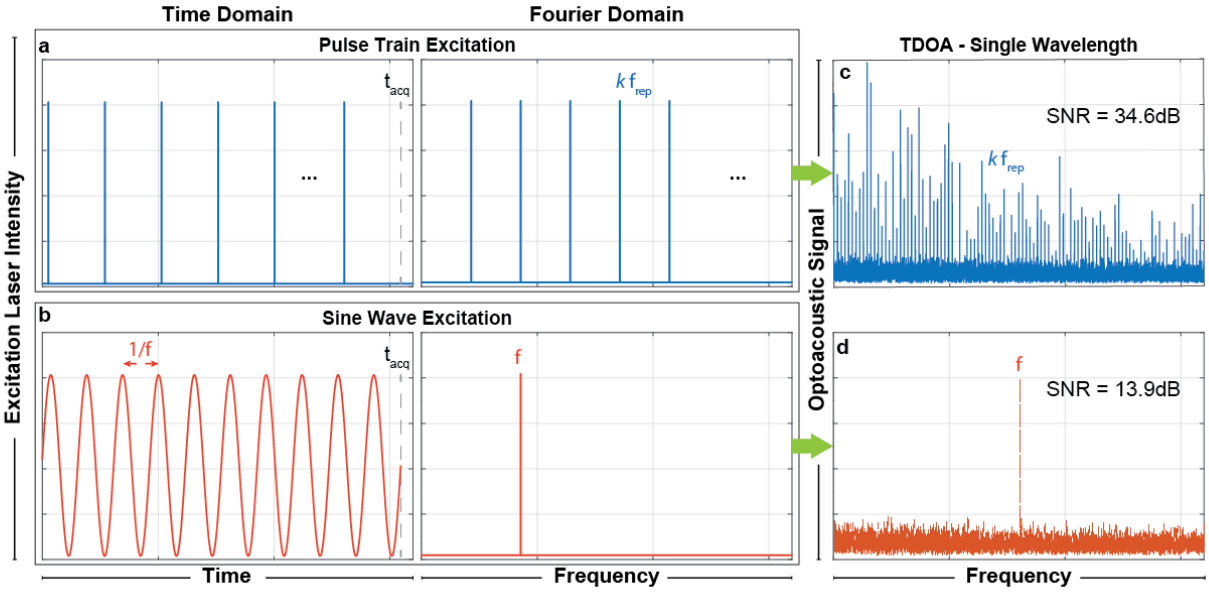

Supplementary Figure 1. Frequency Wavelength Multiplexed Optoacoustic Tomography (FWMOT) advantages over FD-OA at single-wavelength illumination. (a) The FWMOT pulse train excitation in time domain and Fourier space. (b) The sinusoidal FD excitation at frequency f in time domain and Fourier space. (c) The OA signal from a black varnish layer on a Petri dish after excitation with the pattern in (a) in the Fourier space after recording it in time and performing the Fourier transform. The signal appears as many discrete frequency peaks that are all harmonics of the laser pulse repetition rate f_{rep}. The total OA Signal-to-Noise Ratio (SNR) is calculated to be 34.6 dB. (d) The OA signal from the sinusoidal excitation in figure (b) in Fourier space from the same sample. Now the signal appears in only 1 frequency, f, with an SNR of 13.9 dB.

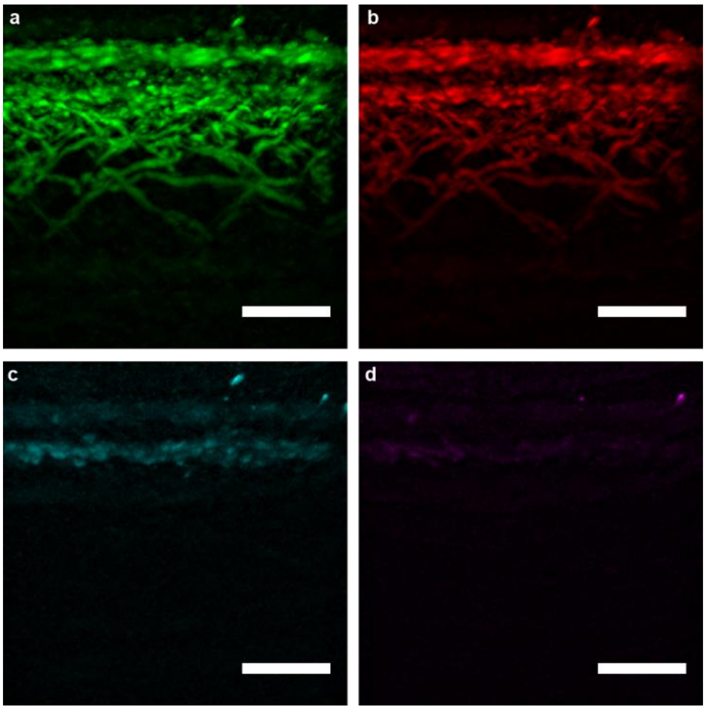

Supplementary Figure 2. Cross-sectional in vivo images of human skin using FCOT at four wavelengths. Figure (a) presents the human skin at 445 nm and (b) at 465 nm. Both wavelengths were able to detect melanin and blood vessels located deeper in the skin. Figure (c) presents the skin at 638 nm where only weak contrast from melanin is detected. Figure (d) shows the skin as detected at 808 nm where blood and melanin absorption are too weak to generate a detectable OA signal. Green is 445 nm, red is 465 nm, purple is 638 nm, cyan is 808 nm, scale bar 1 mm in the horizontal direction, vertical range is 1 mm total. This experiment was performed once.

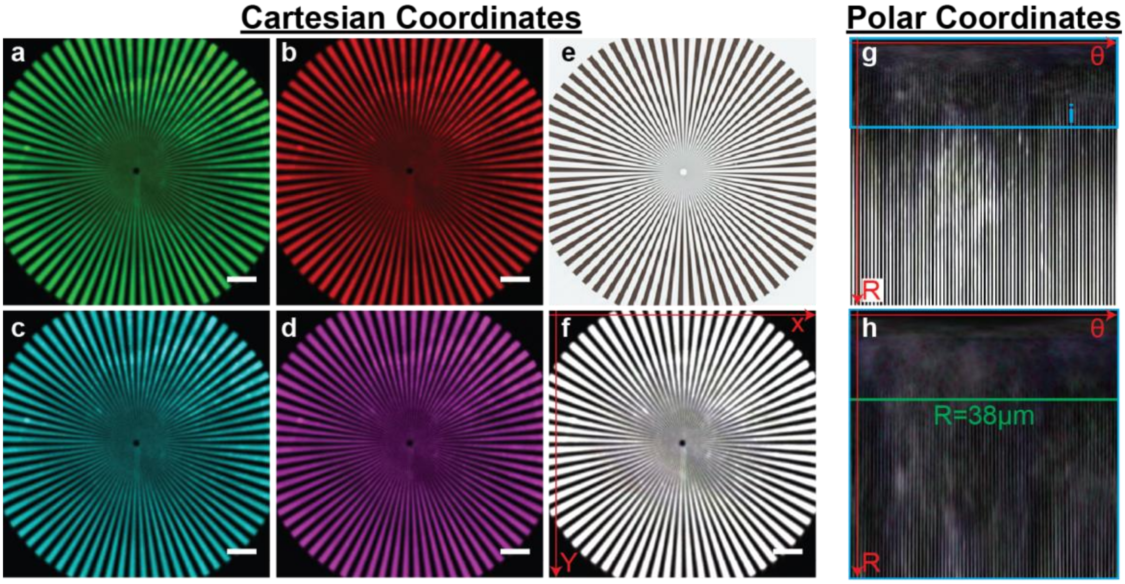

Supplementary Figure 3. OA images from the Siemens Star Resolution phantom. (a-d) The maximum intensity projections of the reconstructed OA images in Cartesian coordinates from wavelengths 445, 465, 638, and 808 nm, respectively. (e) The bright field image of the original Siemens Star phantom. (f) The composite OA image of the same phantom in Cartesian coordinates. All scale bars are 1 mm wide. (g) The composite OA image from figure (f) presented in polar coordinates. (h) A zoomed-in image from the cyan box marked with 'i' in figure (g). The green line indicates the smallest radius that all lines can be well resolved which results in a spatial resolution of 38 µm.

Supplementary Note 1: Frequency Multiplexed Algorithm – Formula derivation

In FWMOT the recorded signal over N_{p} pulses is $S_{\mathrm{r}}(t) = S(t) + n(t)$, where $S(t)$ is the periodic OA signal and $n(t)$ the additive white Gaussian noise. Since $S(t)$ is periodic, we can express its k-th period as $S_{\mathrm{r,k}}(t) = S_{\mathrm{r}}(t) * \delta(t + kT)$, where $*$ denotes the convolution with the delta function. From Fourier theory we know that

$$S(\omega) = S_0(\omega) \sum_{m=-\infty}^{\infty} \delta(\omega - m\omega_0), \tag{1}$$

with $\omega_0 = 2\pi/T$ and T the period of $S_{\mathrm{r}}(t)$. This equation indicates that the Fourier transform of a periodic signal is the Fourier transform over one period of the periodic signal multiplied by a series of deltas that confine the Fourier transform of the periodic signal to the harmonics of its repetition rate. We can now calculate the averaged signal

$$S_{\mathrm{a}}(t) = \frac{1}{N_{\mathrm{p}}} \sum_{k=1}^{N_{\mathrm{p}}} S_{\mathrm{r,k}}(t) = \frac{1}{N_{\mathrm{p}}} \sum_{k=1}^{N_{\mathrm{p}}} S_{\mathrm{r}}(t) * \delta(t + kT) \tag{2}$$

by shifting the recorded signal back by a multiple of the period each time and summing, with t being $0 < t < T$. We can now apply the Fourier transform in equation (2):

$$S_{\mathrm{a}}(\omega) = \frac{1}{N_{\mathrm{p}}} \sum_{k=1}^{N_{\mathrm{p}}} S_{\mathrm{r}}(\omega)\, e^{jk\omega T} = \frac{1}{N_{\mathrm{p}}} S_{\mathrm{r}}(\omega) \sum_{k=1}^{N_{\mathrm{p}}} e^{jk\omega T} \tag{3}$$

Since we know that $S_{\mathrm{r}}(t)$ contains a periodic signal with additive noise we can restrict it to the harmonics of the periodic signal, i.e., multiply equation (3) by $\sum_{m=-\infty}^{\infty} \delta(\omega - m\omega_0)$:

$$S_{\mathrm{a}}(m\omega_0) = \frac{1}{N_{\mathrm{p}}} S_{\mathrm{r}}(\omega) \sum_{k=1}^{N_{\mathrm{p}}} e^{jk\omega T} \sum_{m=-\infty}^{\infty} \delta(\omega - m\omega_0). \tag{4}$$

However, $e^{ikm\omega_0 T} = e^{ikm2\pi} = 1$ for every integer k and m. Therefore, the sum of the exponentials is N_{p} and equation (4) becomes

$$S_{\mathrm{a}}(m\omega_0) = S_{\mathrm{r}}(\omega) \sum_{m=-\infty}^{\infty} \delta(\omega - m\omega_0). \tag{5}$$

This equation tells us that the Fourier transform of the averaged signal is the Fourier transform of the recorded signal confined to the harmonics of its repetition rate. We can further expand the recorded signal to obtain

$$S_{\mathrm{a}}(m\omega_0) = S(\omega) \sum_{m=-\infty}^{\infty} \delta(\omega - m\omega_0) + n(\omega) \sum_{m=-\infty}^{\infty} \delta(\omega - m\omega_0) = S_0(m\omega_0) + n(m\omega_0), \tag{6}$$

which tells us that the Fourier transform of the averaged signal is the Fourier transform of the first period of the periodic signal, what we wanted to recover, plus the Fourier transform of the white Gaussian noise confined to the harmonics of the repetition rate of the periodic signal. Since it is not periodic, the noise is significantly suppressed with its power reduced by $\sqrt{N_{\mathrm{p}}}$.

Supplementary Note 2: Modulation parameters for the FWMOT – Formula Derivation

In this work, the FWMOT signal is acquired in the time domain using a high speed digitizer. In FWMOT the N wavelengths have a different repetition rate, with a small frequency shift $\delta f << f_{\mathrm{rep}}$. For each wavelength, we follow the same FWMOT signal processing algorithm for a single wavelength, choose only the harmonics of the laser repetition rate and apply the inverse Fourier transform to recover the TD signal. For the case of multiple wavelengths, all harmonics from any laser repetition rate should be accurately resolved from all harmonics of each other's laser repetition rate. If any of the harmonics are not well-resolved, there will be a cross-talk between the signals from different wavelengths. Therefore, the choice of the number of pulses in the pulse train and the frequency shift δf is critical to be able to separate the signal from each wavelength. In the following we present the analytical derivation of the formulas that impose the limits on the number of pulses in the pulse train for each wavelength and the frequency shift.

The first wavelength has a reference repetition rate $f_{\mathrm{rep,1}}$, the second wavelength a repetition rate $f_{\mathrm{rep,2}} = f_{\mathrm{rep,1}} + \delta f$ and the Nth wavelength a repetition rate $f_{\mathrm{rep,N}} = f_{\mathrm{rep,1}} + (N-1) * \delta f$. The pulse train of the first laser contains N_{pulses} pulses; this leads to $t_{acq} = N_{pulses}/f_{rep1}$ acquisition time. For each AScan we apply a Fourier transform on the data to get the discrete frequency peaks in the Fourier domain. In Fourier domain, the maximum frequency resolution will be $df = 1/t_{\mathrm{acq}}$. The Ultrasound Transducer (UST) exhibits a specific detection frequency range from f_{low} tof_{high}, taken as the -6 dB cut-off point of its frequency response.

For the reference wavelength, laser 1, we need to find the harmonics that are located and the limits of the UST detection range, i.e., the first k_{s} for which, $k_{\mathrm{s}} * f_{\mathrm{rep,1}} > f_{\mathrm{low}}$, and the last k_{f} for which, $k_{\mathrm{f}} * f_{\mathrm{rep,1}} < f_{\mathrm{high}}$. This happens for k_{s} =ceil($f_{\mathrm{low}}/f_{\mathrm{rep,1}}$) and k_{f} =floor($f_{\mathrm{high}}/f_{\mathrm{rep,1}}$), where floor($x$) rounds x to the nearest integer smaller than or equal to x and ceil(x) rounds x to the nearest integer greater or equal than x.

At the lower end of the UST detection range, the harmonic k_{s} of laser 1 will be very close to the k_s harmonic of laser 2, i.e., $\Delta f = k_{\mathrm{s}} * f_{\mathrm{rep,2}} - k_{\mathrm{s}} * f_{\mathrm{rep,1}} = k_{\mathrm{s}} * (f_{\mathrm{rep,1}} + \delta f) - k_{\mathrm{s}} * f_{\mathrm{rep,1}} = k_{\mathrm{s}} * \delta f$. To be able to resolve these two frequencies $\Delta f > df$, i.e. $\delta f > df/k_{\mathrm{s}}$. This condition applies a minimum limit to the frequency shift δf, with $\delta f_{\mathrm{min}} = df/k_{\mathrm{s}}$.

At the higher end of the UST detection range, the k_f harmonic of laser 1 will be very close to the $k_f - 1$ harmonic of laser N, i.e. $\Delta f = k_{\mathrm{f}} * f_{\mathrm{rep,1}} - (k_{\mathrm{f}} - 1) * f_{\mathrm{rep,N}} = k_{\mathrm{f}} * f_{\mathrm{rep,1}} - (k_{\mathrm{f}} - 1) * [f_{\mathrm{rep,1}} + (N-1) * \delta f] = f_{\mathrm{rep,1}} - (k_{\mathrm{f}} - 1) * (N-1) * \delta f$. To be able to resolve these two frequencies $\Delta f > df$, i.e. $\delta f < (f_{\mathrm{rep,1}} - df)/[(k_{\mathrm{f}} - 1) * (N-1)]$. This condition applies a maximum limit to the frequency shift δf, with $\delta f_{\mathrm{max}} = (f_{\mathrm{rep,1}} - df)/[(k_{\mathrm{f}} - 1) * (N-1)]$.

We observe that as N_{pulses} decreases, the acquisition time decreases and the frequency resolution df increases. Therefore, as N_{pulses} decreases, δf_{min} increases and δf_{max} decreases. As δf_{min} should be smaller than δf_{max}, this imposes a minimum limit for N_{pulses} in the case that δf_{min}=δf_{max}, $N_{\text{pulses\_min}} = \text{ceil}\{[(k_{\text{f}} - 1) * (N - 1) + k_{\text{s}}]/k_{\text{s}}\}$. Any number of pulses in the pulse train larger than $N_{\text{pulses\_min}}$ is valid and allows for a valid definition of a range for the small frequency shift δf, between δf_{min} and δf_{max}.

The minimum number of pulses depends on the reference repetition rate, $f_{\text{rep,1}}$, the UST detection range, f_{low} and f_{high}, and the number of wavelengths used, N. Once a valid N_{pulses} is chosen, the range of valid δf's then depends on $f_{\text{rep,1}}$, f_{low}, f_{high}, N and N_{pulses} and a valid frequency shift can be chosen.

Following the above, for the bandwidth of the UST that we used (22-78 MHz) we use a reference repetition rate of $f_{\text{rep,1}}$=200 KHz and 4 wavelengths. This results in a minimum possible N_{pulses} equal to 12. Since we are using laser diodes that usually provide low SNR, we chose N_{pulses}=100 to increase the SNR through averaging. For this number of pulses, δf can be in the range between 18.2 Hz and 169.7 Hz. For the frequency shift we chose δf=125 Hz, which gives the base repetition rate for each laser as $f_{\text{rep,1}}$=200000 Hz, $f_{\text{rep,2}}$=200125 Hz, $f_{\text{rep,3}}$=200250 Hz, $f_{\text{rep,4}}$=200375 Hz. Moreover, the number of pulses in each pulse train is adjusted to fit as many pulses as possible in the acquisition time defined by the reference repetition rate and N_{pulses1}. In this case, the following N_{pulses} are chosen for each wavelength respectively, N_{pulses1}=100, N_{pulses2}=101, N_{pulses3}=101, N_{pulses4}=101.

Supplementary Note 3: LD Excitation and OA signal Time Series and Power Spectrum

Supplementary Figure 4 presents the LD Excitation Time Series for FWMOT using 4 wavelengths, with $f_{\text{rep,1}}$=200000Hz and δf=125Hz with N_{avg}=200. Figure S4.a presents the full time series, which displays how each wavelength has a slightly different repetition rate. Fig S4.b presents the 4 first pulses of the pulse train. The LD pulses of L1 and L2 are very close to each other and cannot be separated at this

early stage. The same is true for the LD pulses of L3 and L4, only that there is time delay between L1-L2 and L3-L4. The delay is there because L1 and L2 are triggered by the same AWG (AWG1), which is triggered by the stage or the PC. However, L3 and L4 are triggered by a second AWG (AWG2), which is triggered by AWG1, introducing a small time shift. Fig S4.c shows the 16th pulse of each pulse train. The pulses of each distinct LD are now visible. Fig S4.d show the last 4 pulses of each pulse train, where slightly different periods of each LD are apparent.

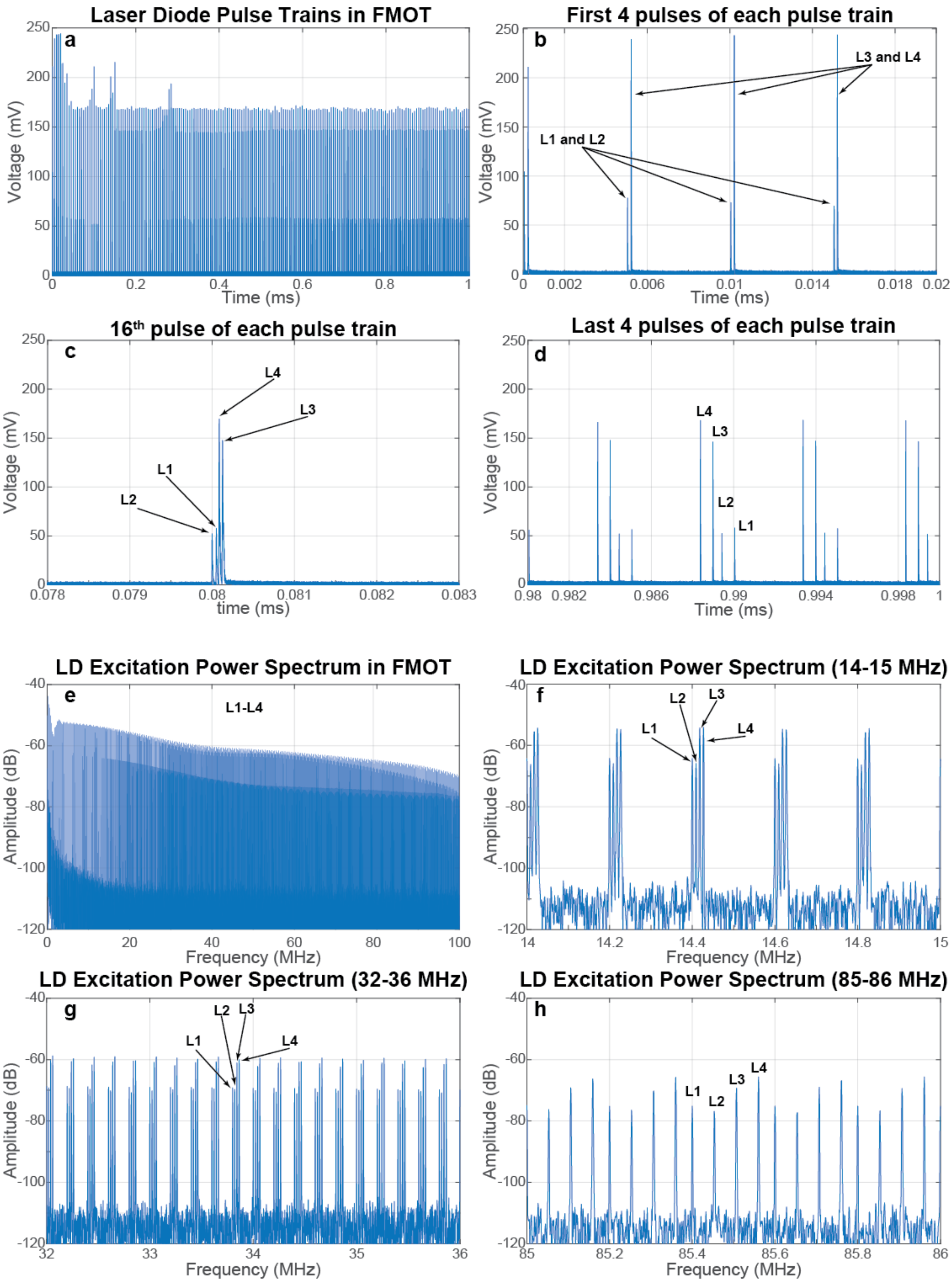

Supplementary Figure 4. The LD excitation time series and power spectrum in FWMOT for four wavelengths. (a) The laser diode excitation time series including all pulses. (b) A zoomed-in plot of the first four pulses. Figure (c) shows the 16th pulse and figure (d) presents the last four pulses of each pulse train. It is apparent that each laser diode pulse has a slightly different period/repetition rate to each other. Figure (e) demonstrates the power spectrum of figure (a). A zoomed-in plot of the power spectrum is presented in figure (f,g,h) for the frequency range between 14-15, 32-36, and 85-86 MHz, respectively. It is also here apparent that the harmonics of each laser can be separated and that each laser has a slightly different repetition rate compared to each other.

Fig S4.e demonstrates the power spectrum of the LD pulse train (Fig S4.a) between 0-100 MHz. Here, a small shift between the harmonics of the different lasers can be observed. Fig S4.f present the power spectrum at the lower end of the UST bandwidth, between 14-15 MHz; the harmonics of each LD can be distinguished. Fig S4.g shows the power spectrum near the center of the UST bandwidth, between 32-36 MHz and Fig S4.h at the upper end of the UST bandwidth, between 85-86 MHz, where the harmonics of each LD are spread further apart, as expected.

We then used the above LD excitation series to generate and detect an OA signal from a black varnish layer on a Petri dish. The resulting time series and power spectrum of the generated OA signal are presented in Fig S5 for the same time points (Fig S5.(a-d) correspond to Fig S4.(a-d)) and frequency range (Fig S5.(e-h) correspond to Fig S5.(e-h)), respectively. We observe that the laser trigger interference appears at the same time points as the laser pulse; the OA signal is expected later in time as it has to travel from the sample to the UST. The OA signal is generated at the excitation frequencies and by choosing the correct set of harmonics for each laser, which can be decomposed to frequency multiplexed signals.

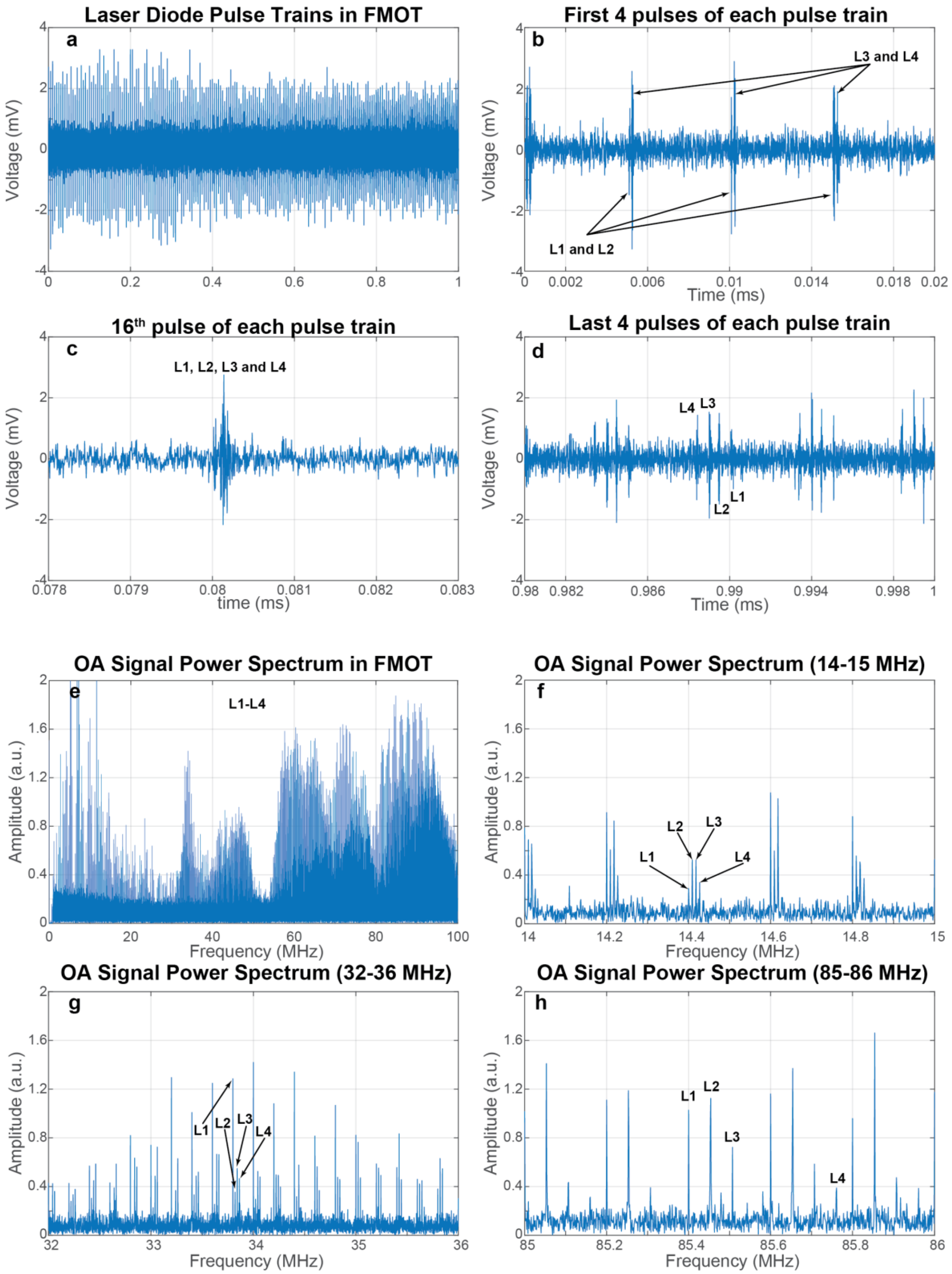

Supplementary Figure 5. The OA Signal time series and power spectrum in FWMOT for four wavelengths from a black varnish target. (a) The OA signal excitation time series including all pulses. (b) A zoomed-in plot of the signal from the first four pulses, figure (c) shows the signal of the 16th pulse and figure (d) presents the signal from the last four pulses of each pulse train. Figure (e) demonstrates the power spectrum of figure (a). A zoomed-in plot of the power spectrum is presented in figure (f,g,h) for the frequency range between 14-15, 32-36, and 85-86 MHz, respectively. It is apparent that the harmonics of the OA signal from each laser can be separated and that each OA signal has a slightly different repetition rate compared to each other.

Supplementary Note 4: Wavelength limit in FWMOT compared to TD OA

There is a maximum number of wavelengths that can be pulsed simultaneously in FWMOT. In all cases, $\delta f_{\min}$ should be smaller than $\delta f_{\max}$, i.e. $\delta f_{\min} < \delta f_{\max}$, and by substituting their values as calculated above and solving for the maximum number of wavelength $N_{\max}$ we get $N_{\max} = \text{floor}\{[(f_{\text{rep},1} - df) * k_{\text{s}}]/[(k_{\text{f}} - 1) * df]\ + 1\}$. Assuming an $f_{\text{rep},1}$=200 kHz, N_{p} =100 and a -6 dB UST bandwidth between 22 and 78 MHz (f_{low}=22 MHz and f_{high}=78 MHz), we get $N_{\max}$=28. For the same parameters in TD OA, a repetition rate of 200 kHz results in a total DoV of 7.5 mm, and assuming that we need at least 1.5 mm DoV for each wavelength, no more than 7.5/1.5=5 wavelengths can be multiplexed without compromising SNR or the total acquisition time.

Supplementary Note 5: Supplementary Information Inventory

The data for this study have been deposited in the Zenodo database under accession code: https://doi.org/10.5281/zenodo.6770729.

All the files are saved as .mat files, the Matlab data file type. The name of each file begins with an "F*x*_" where *x* denotes the Figure these data correspond to.

File "*F1_Data.mat*" contains the one-dimensional data that are presented in Figure 1 using the FWMOT algorithm to benchmark against the time averaging of the same data.

Files "*F2_target_200kHz_xxxAvg_yyy_Lz.mat*" contain the data that are presented in Figure 2. The target can be either Black_Varnish or Noise referring to the acquisition of the Optoacoustic signal from a black varnish layer on a petri dish or the acquisition of Noise. The xxx (200 or 50) value refers to the number of pulses per wavelength as described in the manuscript when referring to Figure 2. The yyy can be either FCOM or DoV referring to the use of the FWMOA algorithm or the Time domain averaging respectively. Lastly, the z value (1-4) refers to the laser used to excite the phantom; in this

case only this wavelength was on during the experiment. When the value "*Lz*" is "*All*", this means that all wavelengths were on and recorded at the same time.

Files "*F3abc_Lx_Measurement1.mat*" are the raw optoacoustic data as they were recorded for each wavelength *x* (1-4) for the mouse ear images presented in Figure 3a-c. Files "*F3defg_Lx_Measurement1.mat*" are the raw optoacoustic data as they were recorded for each wavelength *x* (1-4) for the mouse ear images presented in Figure 3d-g. The *"F3defg_Mouse_Ear_Bright_Field.jpg*" is the bright field image of the mouse ear for the same experiment presented in F3h.

Files "*F4_Lx_Measurement2.mat*" are the raw optoacoustic data as they were recorded for each wavelength *x* (1-4) for the mouse ear images presented in Figure 4a. Files "*F4_Lx_BScans_Measurement4.mat*" are the raw Bscan data as they were recorded for each wavelength x (1-4) for the oxygen stress test presented in Figure 4b-c. Files "*F4_Lx_BScans_Measurement5.mat*" are the raw Bscan data as they were recorded for each wavelength x (1-4) for the ICG and Evan's Blue intravascular injection presented in Figure 4d.

Files "*F6_Lx_Human_Left_Lower_arm.mat*" are the raw optoacoustic data as they were recorded for each wavelength *x* (1-4) for the human skin experiment in Supplementary Figure 3.
