# Supplementary material for: Frequency wavelength multiplexed optoacoustic tomography
Source: Nat Commun. 2022 Aug 1;13:4448. doi: 10.1038/s41467-022-32175-6 (PMC9343396; doi:10.1038/s41467-022-32175-6)
Supplement: Supplementary file 3 — Reporting Summary [file 41467_2022_32175_MOESM3_ESM.pdf]

nature portfolio

Corresponding author(s): Vasilis Ntziachristos

Last updated by author(s): Jun 30, 2022

Reporting Summary

Nature Portfolio wishes to improve the reproducibility of the work that we publish. This form provides structure for consistency and transparency in reporting. For further information on Nature Portfolio policies, see our Editorial Policies and the Editorial Policy Checklist.

Statistics

For all statistical analyses, confirm that the following items are present in the figure legend, table legend, main text, or Methods section.

n/a	Confirmed	
☒	☐	The exact sample size (*n*) for each experimental group/condition, given as a discrete number and unit of measurement
☒	☐	A statement on whether measurements were taken from distinct samples or whether the same sample was measured repeatedly
☒	☐	The statistical test(s) used AND whether they are one- or two-sided<br>*Only common tests should be described solely by name; describe more complex techniques in the Methods section.*
☒	☐	A description of all covariates tested
☒	☐	A description of any assumptions or corrections, such as tests of normality and adjustment for multiple comparisons
☐	☒	A full description of the statistical parameters including central tendency (e.g. means) or other basic estimates (e.g. regression coefficient) AND variation (e.g. standard deviation) or associated estimates of uncertainty (e.g. confidence intervals)
☒	☐	For null hypothesis testing, the test statistic (e.g. *F*, *t*, *r*) with confidence intervals, effect sizes, degrees of freedom and *P* value noted<br>*Give P values as exact values whenever suitable.*
☒	☐	For Bayesian analysis, information on the choice of priors and Markov chain Monte Carlo settings
☒	☐	For hierarchical and complex designs, identification of the appropriate level for tests and full reporting of outcomes
☒	☐	Estimates of effect sizes (e.g. Cohen's *d*, Pearson's *r*), indicating how they were calculated

Our web collection on statistics for biologists contains articles on many of the points above.

Software and code

Policy information about availability of computer code

Data collection	The authors declare that for data collection the commercially available software from GaGe (Compuscope Driver Version 5.04.34, Dynamic Signals LLC, USA) and Matlab 2016b (Matlab, Mathworks, USA) was used.
Data analysis	The authors declare that data analysis was conducted in Matlab 2016b (Matlab, Mathworks, USA) using its built-in functions. The Frequency Wavelength Multiplexed Optoacoustic Tomography algorithm has been patented and is available upon discretion from the corresponding author.

For manuscripts utilizing custom algorithms or software that are central to the research but not yet described in published literature, software must be made available to editors and reviewers. We strongly encourage code deposition in a community repository (e.g. GitHub). See the Nature Portfolio guidelines for submitting code & software for further information.

Data

Policy information about availability of data

All manuscripts must include a data availability statement. This statement should provide the following information, where applicable:

- Accession codes, unique identifiers, or web links for publicly available datasets
- A description of any restrictions on data availability
- For clinical datasets or third party data, please ensure that the statement adheres to our policy

The raw optoacoustic signal data for validating the Frequency Wavelength Multiplexed Optoacoustic Tomography algorithm, the raw imaging data of mice and

humans, the raw B-Scan data of the Oxygen stress test and ICG and Evan's Blue injection experiments that were generated in this study have been deposited in the Zenodo database under accession code: https://doi.org/10.5281/zenodo.6770729

Human research participants

Policy information about studies involving human research participants and Sex and Gender in Research.

Reporting on sex and gender	The authors declare that one human volunteer took part in this study (healthy, white, male, 30 years old).
Population characteristics	The authors declare that one human volunteer took part in this study (healthy, white, male, 30 years old).
Recruitment	The healthy human volunteer was one of the authors.
Ethics oversight	After consultation with the TUM Ethics Commission, no formal ethics approval was necessary. Informed consent form is archived.

Note that full information on the approval of the study protocol must also be provided in the manuscript.

Field-specific reporting

Please select the one below that is the best fit for your research. If you are not sure, read the appropriate sections before making your selection.

☒ Life sciences ☐ Behavioural & social sciences ☐ Ecological, evolutionary & environmental sciences

For a reference copy of the document with all sections, see nature.com/documents/nr-reporting-summary-flat.pdf

Life sciences study design

All studies must disclose on these points even when the disclosure is negative.

Sample size	No sample size calculation was performed because the biological samples (two mice and one human volunteer were imaged in this study) were used only as a vehicle to demonstrate a novel imaging approach.
Data exclusions	The authors declare that no data have been excluded form the analysis.
Replication	The experiments for Figure 3 were replicated independently ten times and the experiments for Figure 4 three times with similar results.
Randomization	Randomization was not relevant to our study because no treatments were involved.
Blinding	Blinding was not relevant to our study because there was only one experimental group.

Reporting for specific materials, systems and methods

We require information from authors about some types of materials, experimental systems and methods used in many studies. Here, indicate whether each material, system or method listed is relevant to your study. If you are not sure if a list item applies to your research, read the appropriate section before selecting a response.

Materials & experimental systems

n/a	Involved in the study	
☒	☐	Antibodies
☒	☐	Eukaryotic cell lines
☒	☐	Palaeontology and archaeology
☐	☒	Animals and other organisms
☒	☐	Clinical data
☒	☐	Dual use research of concern

Methods

n/a	Involved in the study	
☒	☐	ChIP-seq
☒	☐	Flow cytometry
☒	☐	MRI-based neuroimaging

Animals and other research organisms

Policy information about studies involving animals; ARRIVE guidelines recommended for reporting animal research, and Sex and Gender in Research

Laboratory animals	In this study we used 5-6 week old, female Athymic nude-Foxn1 mice. The mice were maintained in an individual ventilated cage system (Tecniplast, Germany) at 22° ambient temperature, relative humidity ~50%, and regular 12h day/night cycle in our specific-pathogen-free (SPF) mouse facility
Wild animals	The study did not involve wild animals.
Reporting on sex	In this study we used 5-6 week old, female Athymic nude-Foxn1 mice.
Field-collected samples	The study did not involve field-collected samples.
Ethics oversight	All procedures involving animal experiments were approved by the Government of Upper Bavaria.

Note that full information on the approval of the study protocol must also be provided in the manuscript.
